# Immunogenicity and Safety of an Inactivated Enterovirus 71 Vaccine Administered Simultaneously with Hepatitis B Virus Vaccine, Group A Meningococcal Polysaccharide Vaccine, Measles-Rubella Combined Vaccine and Japanese Encephalitis Vaccine: A Multi-Center, Randomized, Controlled Clinical Trial in China

**DOI:** 10.3390/vaccines10060895

**Published:** 2022-06-02

**Authors:** Xiaodong Liu, Shaoying Chang, Ruize Wang, Yanhui Xiao, Fangjun Li, Qing Xu, Shaobai Zhang, Xiao Chen, Shangxiao Zhang, Min Zhang, Xiaoqi Chen, Qingfan Cao, Xiaoyu Liu, Hui Wang, Daihong Zhan, Haiping Chen, Wei Chen, Jianyong Jiang, Chao Zhang, Haijiao Wang, Lidong Gao, Xuanwen Shi, Xiaoming Yang, Aiqiang Xu

**Affiliations:** 1 Shandong Provincial Center for Disease Control and Prevention, Jinan 250014, China; liuxd1983@126.com (X.L.); xqepi@163.com (Q.X.); 2 Academy of Preventive Medicine, Shandong University, Jinan 250100, China; 3 Shanxi Provincial Center for Disease Control and Prevention, Taiyuan 030012, China; chshy007@163.com (S.C.); yinyizhe2@163.com (X.C.); wanghui274@163.com (H.W.); haijiao_wang@163.com (H.W.); 4 Shaanxi Provincial Center for Disease Control and Prevention, Xi’an 710054, China; zxfzhk@126.com (R.W.); maolyzhang@163.com (S.Z.); liuxiaoyu594230@163.com (X.L.); zhangchao3759@126.com (C.Z.); 5 China National Biotec Group Company Limited, Beijing 100024, China; xiaoyanhui@sinopharm.com (Y.X.); zhangmin26@sinopharm.com (M.Z.); chenhaiping@sinopharm.com (H.C.); shixuanwen@sinopharm.com (X.S.); 6 Hunan Provincial Center for Disease Control and Prevention, Changsha 410005, China; ymlc05@hncdc.com (F.L.); ymlc06@hncdc.com (S.Z.); gaolidong@hncdc.com (L.G.); 7 Wuhan Institute of Biological Products Company Limited, Wuhan 430200, China; chenxiaoqi@sinopharm.com (X.C.); chenwei17@sinopharm.com (W.C.); 8 Rushan City Center for Disease Control and Prevention, Rushan 264500, China; jhmyrushan@163.com; 9 Shimen County Center for Disease Control and Prevention, Changde 415300, China; zdhlwj@163.com; 10 Haiyang City Center for Disease Control and Prevention, Haiyang 265199, China; 1973jjy@163.com

**Keywords:** inactivated enterovirus 71 vaccine, simultaneous administration, hand, foot and mouth disease, separate administration

## Abstract

Background: The aim of this study was to investigate the immunogenicity and safety of the enterovirus 71 vaccine (EV71 vaccine) administered alone or simultaneously. Methods: A multi-center, open-label, randomized controlled trial was performed involving 1080 healthy infants aged 6 months or 8 months from Shandong, Shanxi, Shaanxi, and Hunan provinces. These infants were divided into four simultaneous administration groups and EV71 vaccine separate administration group. Blood samples were collected from the infants before the first vaccination and after the completion of the vaccination. This trial was registered in the Clinical Trials Registry (NCT03519568). Results: A total of 895 were included in the per-protocol analysis. The seroconversion rates of antibodies against EV71 in four simultaneous administration groups (98.44% (189/192), 94.57% (122/129), 99.47% (187/188) and 98.45% (190/193)) were non-inferior to EV71 vaccine separate administration group (97.93% [189/193]) respectively. Fever was the most common adverse event, the pairwise comparison tests showed no difference in the incidence rate of solicited, systemic or local adverse events. Three serious adverse events related to the vaccination were reported. Conclusions: The evidence of immunogenicity and safety supports that the EV71 vaccine administered simultaneously with vaccines need to be administered during the same period of time recommended in China.

## 1. Introduction

Hand, foot, and mouth disease (HFMD) is a common childhood disease characterized by a brief febrile illness, typical vesicular rashes on the palms, soles, or buttocks, and oropharyngeal ulcers, which is mainly caused by coxsackievirus A16 (CA16) and enterovirus type 71 (EV71). Outbreaks occurred in the Southeast and East Asian regions, since the first outbreak of HFMD have been reported in Canada in 1957 [1,2,3,4,5,6], among which, approximately 500,000 infections and 126 deaths in infants and young children in China in 2008 [5]. The majority of severe and fatal HFMD cases are caused by EV71 [7], and there is no effective treatment for EV71 infection at present, highlighting the need for an effective EV71 vaccine [8].

Three inactivated EV71 vaccines for HFMD among children have been approved by the Chinese Food and Drug Administration and available in China since 2016. All EV71 vaccines showed good safety, immunogenicity, effectiveness, and immunity persistence based on the clinical trials data [9,10,11,12,13,14]. Furthermore, real-world evidence of immunization of the EV71 vaccine proved that the average incidence rate of EV71 HFMD was 60% lower than predicted in the absence of immunization in China in 2017–2018 [15]. Considering that two doses of EV71 vaccine were suggested to be administered to infants aged 6 months to 12 months with an interval of 1 month, however, there was a busy immunization schedule recommended by China’s National Immunization Program (NIP) within the first year of life, which included hepatitis B virus vaccine administered at 6 months of age, group A meningococcal polysaccharide vaccine administered at 6 months and 9 months of age, measles-rubella combined vaccine and Japanese encephalitis vaccine administered at 8 months of age, etc. Thus, there is an urgent need to investigate the immunogenicity and safety of the EV71 vaccine administered alone or simultaneously with hepatitis B virus vaccine, group A meningococcal polysaccharide vaccine, measles-rubella combined vaccine and Japanese encephalitis vaccine.

In this study, we conducted a multi-center, randomized-controlled clinical trial to evaluate the immunogenicity and safety of the EV71 vaccine administered alone or simultaneously with four kinds of vaccines that need to be administered during the same period recommended by NIP in 2018.

## 2. Methods

### 2.1. Study Design

An open-label, randomized-controlled, non-inferiority trial was conducted in 4 centers including Shandong province, Shanxi province, Shaanxi province, and Hunan province in 2018. This study was approved by the ethics committees of the Shandong Provincial Center for Disease Control and Prevention, Shanxi Provincial Center for Disease Control and Prevention, Shaanxi Provincial Center for Disease Control and Prevention, and Hunan Provincial Center for Disease Control and Prevention. This study was registered at clinicaltrial.gov (NCT03519568).

### 2.2. Participants

In accordance with the necessary ethical requirements, informed consent form was signed by the parents or legal guardian of the infant. The parents or legal guardian of the infants were informed that they could withdraw from the study at any time, but the reason for the withdrawal should be collected, if possible.

All enrolled infants must meet the following criteria: 6 months or 8 months of age on the day of enrollment, all scheduled vaccinations according to the national immunization recommendations received, ≥14 days since last immunization, ≤37.0 °C body temperature, and an informed consent form signed by the parents or legal guardian.

The infant was excluded from the study if he or she met one of the following criteria: completion of 3 doses of hepatitis B vaccine administration, prior administration of meningococcal polysaccharide vaccine or measles-containing vaccine or Japanese encephalitis vaccine or EV71 vaccine, history of HFMD, allergy to the active substance, any non-active substance, or the manufacturing process of the vaccine, the presence of a serious chronic disease, allergic constitution, fever, or acute disease.

### 2.3. Vaccine

A licensed EV71 vaccine used in this study was manufactured by the Wuhan Institute of Biological Products Company Limited. The EV71 vaccine was an inactivated EV71 vaccine cultured by Vero cell for proliferation, containing ≥ 3.0 EU of antigen, with a seed virus of EV71 strain AHFY087VP5 (genotype C4, which is the predominant strain in mainland China) [16]. Considering that the third dose of the hepatitis B virus vaccine should be given to the same manufacturer as the first two doses, hepatitis B virus vaccines from three manufacturers were used in this study. Hepatitis B virus vaccine used at the Shandong center was manufactured by Dalian Hissen Bio-pharm Ltd (Dalian, China), used at the Shaanxi center was manufactured by NCPC GeneTech Biotechnology Pharmaceutical Company Limited (Shanxi, China), and used at the Hunan and Shanxi centers were manufactured by Shenzhenkangtai Biotechnology Company Limited (Shenzhen, China), all of which contained 10 µg HBsAg. Group A meningococcal polysaccharide vaccine was manufactured by Wuhan Institute of Biological Products Company Limited (containing 150 µg polysaccharide, for 5 doses, each dose should contain ≥ 30 µg polysaccharide) (Wuhan, China). Measles-rubella combined vaccine was manufactured by Beijing Institute of Biological Products Company Limited (containing ≥ 3.2 LgCCID50 measles, rubella live virus) (Beijing, China). Japanese encephalitis vaccine was manufactured by Chengdu Institute of Biological Products Company Limited (containing ≥ 5.4 LgPFU of live Japanese encephalitis virus) (Chengdu, China).

### 2.4. Randomization and Masking

**Figure 1:**
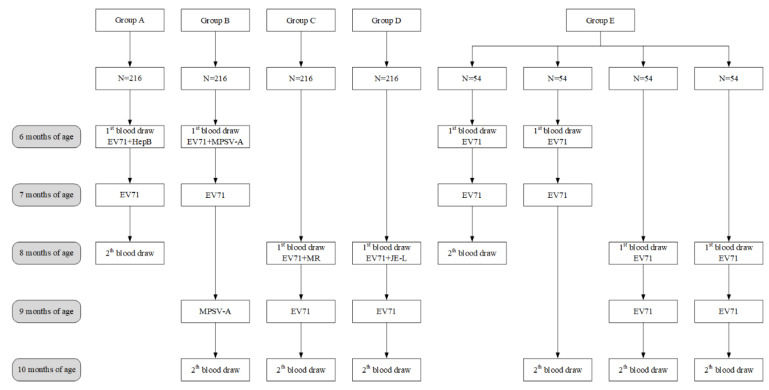
Flowchart of blood drawing and vaccinations in five study groups. Group A: EV71 vaccine and hepatitis B virus vaccine simultaneous administration group; Group B: EV71 vaccine and group A meningococcal polysaccharide vaccine simultaneous administration group; Group C: EV71 vaccine and measles-rubella combined vaccine simultaneous administration group; Group D: EV71 vaccine and Japanese encephalitis vaccine simultaneous administration group; Group E: EV71 vaccine separate administration group. EV71 = Inactivated enterovirus 71 vaccine; HepB = Hepatitis B virus vaccine; MPSV-A = Group A meningococcal polysaccharide vaccine; MR = measles-rubella combined vaccine; JE-L = Live, attenuated Japanese encephalitis vaccine.

A random number table was generated by SAS 9.4, and 1080 random numbers were allocated (1:1:1:1:1) into four simultaneous administration groups and a separate administration group by stratified block randomization. According to the study design of the EV71 vaccine and four kinds of vaccine simultaneous administration, random numbers were divided into four stratifications, in which the age of months for EV71 vaccinations and blood sampling were the same for each stratification, and the random numbers were assigned to the simultaneous administration group and separate administration group using a 4:1 block randomization scheme (Figure 1). The open-label design was used to minimize unnecessary injection; however, the laboratory technicians were blinded to the type of vaccine administered to each group.

### 2.5. Procedures

Four simultaneous administration groups and a separate administration group were established in this study. Group A: EV71 vaccine and HepB vaccine simultaneous administration group, infants were administered with EV71 vaccine (at six months and seven months of age, respectively) and HepB vaccine (at six months of age). Group B: EV71 vaccine and group A meningococcal polysaccharide vaccine simultaneous administration group, infants were administered with EV71 vaccine (at six months and seven months of age, respectively) and group A meningococcal polysaccharide vaccine (at six months and nine months of age, respectively). Group C: EV71 vaccine and measles-rubella combined vaccine simultaneous administration group, infants were administered with EV71 vaccine (at eight months and nine months of age, respectively) and measles-rubella combined vaccine (at eight months of age). Group D: EV71 vaccine and Japanese encephalitis vaccine simultaneous administration group, infants were administered with EV71 vaccine (at eight months and nine months of age, respectively) and Japanese encephalitis vaccine (at eight months of age). Group E: EV71 vaccine separate administration group (at six months and seven months of age, respectively/at eight months and nine months of age, respectively). Blood specimens were collected from the infants before the first vaccination and after completion of the vaccination, and Figure 1 shows the specific months of blood sampling for each group.

### 2.6. Outcomes

All blood specimens were analyzed at the laboratory of the Beijing Center for Disease Control and Prevention. Neutralizing antibody titers against EV71 were measured using a vaccine strain (AHFY087VP5 strain) based cytopathic effect inhibition assay. A titer of ≥ 1:8 was defined as antibody seropositive. The dilution of serum ranged from 1:8 through 1:16,384, and antibody titer was calculated as half of the cut-off value when it is below the cut-off value (1:8). Seroconversion was defined as any of the following: conversion from antibody seronegative to seropositive, or pre-vaccination antibody titer ≥1:8 and a minimum 4-fold increase at post-vaccination.

The seroconversion rates and 95% confidence interval (CI) of the rate differences (difference in the seroconversion rate between the simultaneous administration groups and the separate administration group) were calculated. Non-inferiority was defined as the lower limit of the 95% CI of the rate difference ≥ −10%.

All infants were monitored for 30 min post-vaccination for adverse events (AEs). Parents or legal guardian of participants used diary cards to record the duration and intensity of any local and systemic AEs occurring ≤30 days after each vaccination. As well, the diary cards were checked by safety assessors via face-to-face or telephone to assure completeness and accuracy. The local AEs included redness, induration, and swelling; solicited systemic AEs included fever, diarrhea, vomiting, irritability, anorexia, and allergy.

### 2.7. Statistical Analyses

The sample size was calculated based on the fact that the seroconversion rates for antibody against EV71 in simultaneous administration group were non-inferior to those in the separate administration group. A one-sided α and efficacy power (1-β) were 0.025 and 80%, respectively. The non-inferiority margin was 10%. Assuming that the seroconversion rate was 94% [16], the sample size was calculated as 170 for each group. Assuming a drop-out rate of 20%, the sample size was estimated to be 214 for each group. In addition, by considering factors such as block size and multiple research centers, the sample size was increased to 216 for each group, with a total number of 1080.

Statistical analyses were performed using SAS version 9.4. The immunogenicity evaluation was conducted for the subjects included in the per-protocol analysis. Safety evaluation was conducted for the subject population with at least one vaccination dose and one safety follow-up record. The seropositive rates, seroconversion rates, and the incidence rate of AEs were compared between groups using a Fisher’s exact test (two-tailed) and χ^2^ test (two-tailed). The geometric mean titer (GMT) was compared between groups using analysis of variance and Student’s *t*-test. For multiple comparisons, the Bonferroni correction method was used to adjust the *p*-values corresponding to statistical test.

## 3. Results

**Figure 2:**
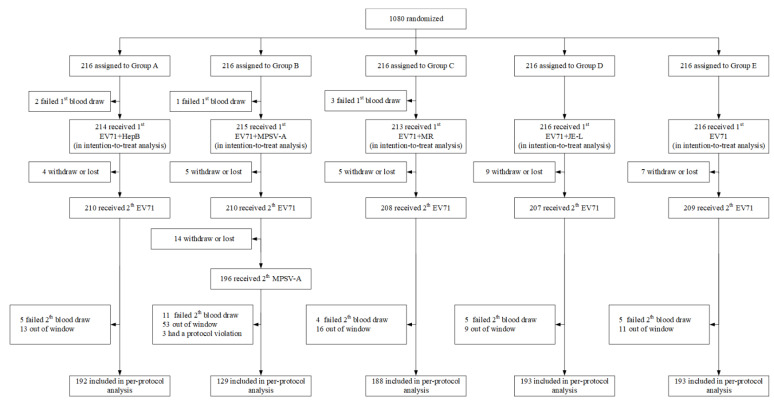
Trial profile, per-protocol analyses (PPS), China, 2018 to 2019.

A total of 1080 infants from four research centers were enrolled in this trial from March 2018 to September 2018 and were randomly allocated into five groups. The per-protocol population was comprised of 895 infants (82.87%), including 192 infants from Group A, 129 infants from Group B, 188 infants from Group C, 193 infants from Group D, and 193 infants from Group E (Figure 2). The characteristics of the infants are shown in Table 1.

Before vaccination, the seropositive rates of antibodies against EV71 were 19.79% (38/192), 18.60% (24/129), 5.85% (11/188), 8.29% (16/193), and 13.47% (26/193) in Group A, Group B, Group C, Group D and Group E, respectively, infants in Group C had lower seropositive rate (5.85% vs. 13.47%, *p* = 0.012); the GMT of antibodies against EV71showed no difference between five groups. (Table 2).

**Figure 3:**
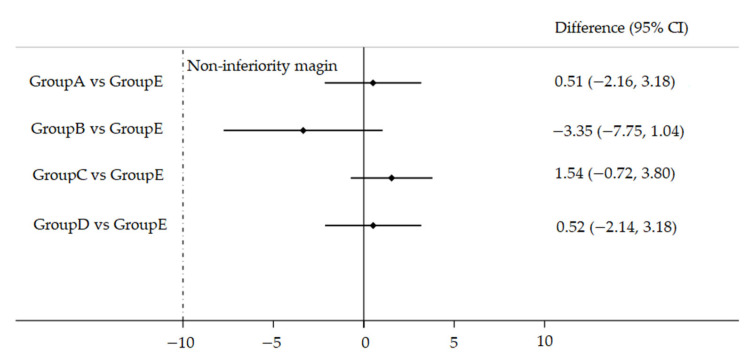
Differences in the proportion of seroconversion for simultaneous administration groups versus separate administration groups, China 2018 to 2019. Differences in the proportion of seroconversion were measured between simultaneous groups (Group A, Group B, Group C, and Group D) and separate group (Group E) with two-sided 95% CIs.

After immunization, seropositive rates of antibodies against EV71 were 99.48% (191/192), 96.12% (124/129), 99.47% (187/188), 99.48% (192/193), and 97.93% (189/193) in Group A, Group B, Group C, Group D and Group E, respectively; seroconversion rates of antibodies against EV71 were 98.44% (189/192), 94.57% (122/129), 99.47% (187/188), 98.45% (190/193) and 97.93% (189/193) in Group A, Group B, Group C, Group D and Group E, respectively, the tests showed no difference in seropositive and seroconversion rates. The seroconversion rates of antibodies against EV71 in simultaneous administration groups (Group A, Group B, Group C, and Group D, respectively) were non-inferior to those in Group E (Figure 3). There was a significant difference in the GMT of antibodies against EV71 for Group A (792.51), Group B (287.93), Group C (680.91), Group D (677.13), and Group E (562.47). Furthermore, based on the pairwise comparison, there was a significant difference in GMT between Group A and Group E, and between Group B and Group E (*p* < 0.0125). (Table 2).

We further analyzed the data of group E (Table 3). According to Figure 1 and Figure 2, the group E (*n* = 193) was divided into four small groups, named group E1 (*n* = 51), group E2 (*n* = 45), group E3 (*n* = 48) and group E4 (*n* = 49) respectively. The seropositive rates and seroconversion rates of antibodies against EV71 showed no difference between the four small groups. There was a significant difference in the GMT of antibodies against EV71 for Group E1 (1004.00), Group E2 (341.06), Group E3 (553.35), and Group E4 (495.08). Furthermore, based on the pairwise comparison, there was a significant difference in GMT between Group E1 and Group E2 (*p* < 0.0167).

The safety evaluation results including the incidence rate of solicited local and systemic AEs and unsolicited AEs were shown in Table 4. The most common AEs consisted of fever, redness, and induration. The incidence rate of solicited AEs was 25.58% (55/215) in Group A, 29.63% (64/216) in Group B, 32.86% (70/213) in Group C, 40.47% (87/215) in Group D, and 32.41% (82/216) in Group E, showed a significant difference between five groups (*p* = 0.019). Similarly, there was a significant difference in the incidence rate of solicited systemic AEs and fever between the five groups, the pairwise comparison tests showed no difference in the incidence rate of solicited AEs, solicited systemic AEs, and fever.

No withdrawal or loss to follow-up due to vaccine-related AEs were observed among the infants who withdrew from the study. One case of serious AE (SAE) was observed that was considered to be related to the vaccine in both Group B (hospitalization due to fever) after the second vaccination, Group C (hospitalization due to diarrhea) after the first vaccination and Group D (hospitalization due to fever) after the first vaccination.

## 4. Discussion

In this study, we found that the seroconversion rates of antibodies against EV71 ranged from 94.57% to 99.47% in five groups after vaccinations, and the seroconversion rates in four simultaneous administration groups were non-inferior to EV71 vaccine separate administration groups, respectively. The safety results demonstrated that the EV71 vaccine administered simultaneously did not increase the incidence of AEs. Three vaccine-related SAEs were reported in simultaneous administration groups, yet none of them dropped out from the study. The high seroconversion rates against EV71 and good safety found in all groups supported the simultaneous administration of EV71 vaccine with hepatitis B virus vaccine, group A meningococcal polysaccharide vaccine, measles-rubella combined vaccine, and Japanese encephalitis vaccine.

Currently, few study data were reported regarding the immunogenicity and safety of the EV71 vaccine administered simultaneously with vaccines that need to be administered during the same period of time. A single-center randomized clinical trial of EV71 vaccine administered simultaneously with hepatitis B virus vaccine and group A meningococcal polysaccharide vaccine conducted in China found that the seroconversion rates of antibodies against EV71 were 98.56% and 98.61% for simultaneous administration group and separate administration group [17]. In addition, a phase IV trial reported the seroconversion rates of antibodies against EV71 was 100% for separate administration of the EV71 vaccine [18]. Although the study design of the trial was different from what we performed in this study, the results of the seroconversion rate were similar to our findings.

An additional finding of our study is that the seropositive rate (96.12% vs. 99.48%, 99.47%, 99.48%, 97.93%), seroconversion rate (94.57% vs. 98.44%, 99.47%, 98.45%, 97.93%) and GMT (287.93 vs. 792.51, 680.91, 677.13, 562.47) for EV71 vaccine and group A meningococcal polysaccharide vaccine simultaneous administration group were the lowest among five groups after vaccination. Further analysis found that the GMT of group E2 was lower, indicating that the reason for the difference was that the second blood sampling of infants who were allocated into group B and group E2 were received at an interval of 3 months after vaccinations of two-dose with EV71, while other groups were performed at an interval of 1 month. A study showed the GMTs were 255.7 and 220.6 at an interval of 2 months, which was consistent with our results [18]. A pevious study proved that the EV71 vaccine could elicit a substantial specific immune response with GMTs of EV71 peaked at 1 month after two-dose vaccination and waned significantly from 1 month through 2 months [9,11].

Of note, our immunogenicity results show that the infants aged 8 months in this study had a lower seropositive rate of antibodies against EV71 than the infants aged 6 months before vaccination. Meta-analysis results found that an average of 78% of neonates were seropositive to EV71 infection, but such maternally conferred immunity almost completely waned by 5 months of age [19]. Thus, it is recommended to administer the EV71 vaccine as early as 6 months of age to prevent HFMD caused by EV71 before the maternal antibody decay to a lower level.

One limitation of our study was that a total of 129 infants were included in per-protocol analysis for the EV71 vaccine and group A meningococcal polysaccharide vaccine simultaneous administration group, which did not meet the expected sample size. Because of the greater number of follow-up times and vaccinations, it resulted in a large number of infants who were out of the window. However, the baseline characteristics, seropositive rate, and GMT for this group were not substantially different from the EV71 vaccine separate administration group among infants who were included in per-protocol analysis; we believe that it is unlikely to bias the immunogenicity and safety results.

In conclusion, our study was the first study to demonstrate that the EV71 vaccine administered simultaneously with hepatitis B virus vaccine, group A meningococcal polysaccharide vaccine, measles-rubella combined vaccine and Japanese encephalitis vaccine was non-inferior to EV71 vaccine administered alone. Therefore, evidence was provided on an available and safe EV71 vaccination strategy that simultaneously administered vaccines need to be administered during the same period of time that is recommended, given the busy vaccination strategy within the first year of life in China.

## Figures and Tables

**Table 1:** Baseline characteristics in total populations and per-protocol populations.

	Group A	Group B	Group C	Group D	Group E
**Total Populations**					
No. of participant	216	216	216	216	216
Age, mean ± SD (months)	6.52 ± 0.02	6.54 ± 0.02	8.56 ± 0.02	8.57 ± 0.02	7.55 ± 0.07
Male sex, *n* (%)	103 (47.69)	103 (47.69)	115 (53.24)	104 (48.15)	110 (50.93)
**Per-Protocol Populations**					
No. of participant	192	129	188	193	193
Age, mean ± SD (months)	6.53 ± 0.02	6.57 ± 0.03	8.56 ± 0.02	8.56 ± 0.02	7.56 ± 0.08
Male sex, *n* (%)	93 (48.44)	59 (45.74)	96 (51.06)	89 (46.11)	100 (51.81)

Group A: EV71 vaccine and hepatitis B virus vaccine simultaneous administration group; Group B: EV71 vaccine and group A meningococcal polysaccharide vaccine simultaneous administration group; Group C: EV71 vaccine and measles-rubella combined vaccine simultaneous administration group; Group D: EV71 vaccine and Japanese encephalitis vaccine simultaneous administration group; Group E: EV71 vaccine separate administration group.

**Table 2:** Antibody responses to EV71 pre- and post-vaccination in the per-protocol populations.

	Group A (*n* = 192)	Group B (*n* = 129)	Group C (*n* = 188)	Group D (*n* = 193)	Group E (*n* = 193)	*p*-Value				
						Five Group	Group A vs. E *	Group B vs. E *	Group C vs. E *	Group D vs. E *
**Pre-Vaccination**										
SPR, *n* (%)	38 (19.79)	24 (18.60)	11 (5.85)	16 (8.29)	26 (13.47)	**0.000**	0.096	0.213	**0.012**	0.102
(95% CI)	(14.73–26.06)	(12.77–26.31)	(3.26–10.28)	(5.13–13.13)	(9.32–19.08)					
GMT	5.06	5.17	4.30	4.61	4.85	0.052				
(95% CI)	(4.55–5.62)	(4.61–5.79)	(4.10–4.51)	(4.16–5.11)	(4.43–5.30)					
**Post-Vaccination**										
SPR, *n* (%)	191 (99.48)	124 (96.12)	187 (99.47)	192 (99.48)	189 (97.93)	0.055				
(95% CI)	(97.13–99.99)	(91.19–98.73)	(97.07–99.99)	(97.15–99.99)	(94.78–99.43)					
SCR, *n* (%)	189 (98.44)	122 (94.57)	187 (99.47)	190 (98.45)	189 (97.93)	0.068				
(95% CI)	(95.50–99.68)	(89.14–97.79)	(97.07–99.99)	(95.52–99.68)	(94.78–99.43)					
GMT	792.51	287.93	680.91	677.13	562.47	**0.000**	**0.007**	**0.000**	0.134	0.166
(95% CI)	(671.95–934.69)	(228.66–362.56)	(576.44–804.30)	(562.35–815.35)	(466.59–678.05)					

* Adjusted *p*-value was 0.0125 (0.05/4).

**Table 3:** Antibody responses to EV71 post- vaccination for group E in the per-protocol populations.

	Group E1 (*n* = 51)	Group E2 (*n* = 45)	Group E3 (*n* = 48)	Group E4 (*n* = 49)	*p*-Value			
					Four Group	Group E1 vs. E2 *	Group E3 vs. E2 *	Group E4 vs. E2 *
**Post-vaccination**								
SPR, *n* (%)	51 (100.00)	43 (95.56)	47 (97.92)	48 (97.96)	0.507			
(95% CI)	(93.02–100.00)	(84.85–99.46)	(88.93–99.95)	(89.15–99.95)				
SCR, *n* (%)	51 (100.00)	43 (95.56)	47 (97.92)	48 (97.96)	0.507			
(95% CI)	(93.02–100.00)	(84.85–99.46)	(88.93–99.95)	(89.15–99.95)				
GMT	1004.00	341.06	553.35	495.08	**0.001**	**0.000**	0.087	0.177
(95% CI)	(738.13–1365.65)	225.06–516.85)	(377.46–811.22)	(343.39–713.77)				

* Adjusted *p* value was 0.0167 (0.05/3).

**Table 4:** Reported adverse events after any vaccination.

Event	Group A (*n* = 215) *n*, (%)	Group B (*n* = 216) *n*, (%)	Group C (*n* = 213) *n*, (%)	Group D (*n* = 215) *n*, (%)	Group E (*n* = 216) *n*, (%)	*p*-Value				
						Five Group	Group A vs. E *	Group B vs. E *	Group C vs. E *	Group D vs. E *
**Total adverse events**	76 (35.35)	83 (38.43)	89 (41.78)	104 (48.37)	82 (37.96)	0.061				
**Solicited adverse events**	55 (25.58)	64 (29.63)	70 (32.86)	87 (40.47)	70 (32.41)	**0.019**	0.118	0.533	0.920	0.082
**Local adverse events**	14 (6.51)	9 (4.17)	2 (0.94)	11 (5.12)	12 (5.56)	0.057				
Redness	8 (3.72)	6 (2.78)	1 (0.47)	7 (3.26)	10 (4.63)	0.127				
Induration	8 (3.72)	6 (2.78)	1 (0.47)	5 (2.33)	5 (2.31)	0.261				
Swelling	3 (1.40)	0 (0)	0 (0)	1 (0.47)	0 (0)	0.074				
**Systemic adverse events**	45 (20.93)	58 (26.85)	69 (32.39)	78 (36.28)	62 (28.70)	**0.007**	0.062	0.667	0.407	0.093
Fever	37 (17.21)	44 (20.37)	58 (27.23)	69 (32.09)	53 (24.54)	**0.003**	0.061	0.299	0.524	0.082
Diarrhea	5 (2.33)	6 (2.78)	5 (2.5)	6 (2.79)	6 (2.78)	0.995				
Vomiting	1 (0.47)	1 (0.46)	0 (0)	0 (0)	1 (0.46)	0.738				
Irritability	0 (0)	1(0.46)	1(0.47)	1 (0.47)	0 (0)	0.733				
Anorexia	1 (0.47)	0 (0)	2 (0.94)	1 (0.47)	0 (0)	0.469				
Allergy	0 (0)	0 (0)	1 (0.47)	1 (0.47)	1 (0.46)	0.733				
**Unsolicited adverse events**	35 (16.28)	34 (15.74)	38 (17.84)	49 (22.79)	28 (12.96)	0.092				

* Adjusted *p* value was 0.0125 (0.05/4).

## Data Availability

Data are available for scientific purposes after a written request to the corresponding author.
